# Evidence of a sylvatic enzootic cycle of *Leishmania infantum* in the State of Amapá, Brazil

**DOI:** 10.1590/0037-8682-0169-2019

**Published:** 2019-12-20

**Authors:** Eduardo Stramandinoli Moreno, Luiz Alberto Sabioni, Marina Maria Moraes de Seixas, Job Alves de Souza, Andreza Pain Marcelino, Paloma Helena Fernandes Shimabukuro

**Affiliations:** 1 Universidade Federal do Oeste do Pará, Programa de Pós-graduação: Sociedade, Natureza e Desenvolvimento, Santarém, PA, Brasil.; 2 Ministério da Saúde, Distrito Sanitário Especial Indígena - Amapá e Norte do Pará, Secretaria Especial de Saúde Indígena, Macapá, AP, Brasil.; 3 Fundação Oswaldo Cruz, Grupo de Estudos em Leishmanioses, Instituto René Rachou, Centro de Referência Nacional e Internacional para Flebotomíneos, Belo Horizonte, MG, Brasil.; 4 Universidade Federal do Amapá, Programa de Pós-graduação em Biodiversidade Tropical, Macapá, AP, Brasil.; 5 Pesquisador independente atualmente não afiliado, Rio de Janeiro, RJ, Brasil.; 6 Fundação Ezequiel Dias, Serviço de Doenças Parasitárias, Belo Horizonte, MG, Brasil.

**Keywords:** Leishmania infantum, Amapá, Enzootic cycle, ITS1, HSP70

## Abstract

**INTRODUCTION:**

*Leishmania infantum* was considered to be absent from Amapá until 2017 when canine infection was detected. However, there is a lack of knowledge about which reservoir species are involved in transmission in this region.

**METHODS:**

Between 2014 and 2016, 86 samples from wild mammals and 74 from domestic dogs were collected in Wajãpi Indigenous Territory and were tested for the presence of deoxyribonucleic acid (DNA) of *Leishmania*.

**RESULTS:**

The DNA of *Le. infantum* was detected in two rodent samples, *Dasyprocta* sp. and *Proechimys cuvieri*.

**CONCLUSIONS:**

This is the first evidence characterizing a sylvatic transmission cycle of *Le. infantum* in the State of Amapá.

American visceral leishmaniasis (AVL) is a zoonotic disease caused by the protozoan parasite *Leishmania (Leishmania) infantum*. In Brazil, AVL has been recorded in all states, except Amazonas and Acre^1^. Autochthonous cases of AVL in the Amazon region have been recorded mainly in the north of the State of Pará and in the State of Roraima, where there have also been reports of the presence of the sand fly species *Lutzomyia longipalpis*, the main vector of *Le. infantum* ^2^. *Lu. longipalpis* was first reported in Amapá in 2013 during environmental assessments undertaken as part of the construction of a hydroelectric power plant in the Municipality of Ferreira Gomes^3^. However, this sand fly species is considered to be either absent or unreported in other regions of this state.

An important wild reservoir host of *Le. infantum*, the crab-eating fox *Cerdocyon thous*, was not reported in Amapá until recently, when it was found in savannas near the municipalities of Ferreira Gomes and Porto Grande (Matapi River) and in the *terra-firme* forest near the Municipality of Macapá^2^. The first case of AVL in a domestic dog in the Municipality of Macapá was recorded in 2017. Since then, AVL was detected in dogs in Macapá and in the Municipality of Mazagão. Despite the occurrence of canine cases and the possible establishment of an urban transmission cycle of AVL, there is a lack of knowledge regarding the sand fly vector and wild reservoir hosts of AVL in the State of Amapá and the Brazilian Amazon biome more generally.

Domestic dogs and several wild mammal species, especially carnivores, rodents, and marsupials, have already been identified as potential reservoir hosts of *Le. infantum* in Brazil and the rest of South America^4^, but no previous studies have identified natural infection in wild vertebrate hosts in the State of Amapá. Here, we present evidence of a sylvatic enzootic transmission cycle of *Le. infantum* in wild rodent hosts in the Municipality of Pedra Branca do Amapari, in the central region of the State of Amapá.

The Wajãpi Indigenous Territory (WIT) is located in the Municipality of Pedra Branca do Amapari, in the State of Amapá, in the eastern region of the Brazilian Amazon, close to the border between Brazil, Suriname, and French Guiana, with a territorial area of 6,070 km^2^ ^5^. To date, no clinical human cases of AVL have been recorded in the WIT. The area is a dense rainforest, with a rugged relief, and belongs one of the world's largest continuous areas of rainforest, covering over 12 million hectares of protected area.

Between 2014 and 2016, we conducted 15 field trips to the WIT, during which eco-epidemiological surveys to study leishmaniasis were carried out in more than 30 villages. The activities were conducted in partnership with the Brazilian Ministry of Health (Secretaria Especial de Saúde Indígena, Distrito Sanitário Especial Indígena Amapá e Norte do Pará). The study was approved by the Comissão Nacional de Ética em Pesquisa (Certificado de Apresentação para Apreciação Ética - CAAE, 20188213.9.0000.5091), Instituto Chico Mendes de Conservação da Biodiversidade (37972-6), and Fundação Nacional do Índio (08620.030843/2014-59).

Regarding potential vertebrate hosts, biological samples from 74 domestic dogs residing in 32 Wajãpi villages were collected during the study. Whole blood samples were collected on filter paper and serum in tubes containing separator gel, while conjunctival swabs were kept in microtubes containing *RNAlater*™ (Sigma-Aldrich). Additionally, biological samples were obtained from animals caught by the Wajãpi as part of their normal subsistence hunting. Skin samples were collected and placed in *RNAlater*™ (Sigma-Aldrich), while coagulated blood was spotted onto filter paper. For each sample, information about the caught animal, the hunter, the environment, and the period of the hunt were recorded. Samples were collected for a period of 547 days from 19 villages.

All the collected samples were sent for laboratory examination, where they were either screened serologically (canine samples) and tested for the presence of DNA (canine and wildlife samples) from *Leishmania* and other trypanosomatids. Serological and molecular tests of canine samples were performed at the Fundação Ezequiel Dias, Belo Horizonte, Minas Gerais, Brazil. Serological tests were performed with immunochromatographic rapid test DPP® (TR DPP/Bio-Manguinhos) and enzyme-linked immunosorbent assay (ELISA) (Bio-Manguinhos), according to the manufacturer’s protocol. Polymerase chain reaction (PCR) was performed on DNA extracted from conjunctival swab of dogs using the QIAamp DNA Mini Kit (Qiagen, Hilden, NW, Germany) and subsequent amplification by conventional PCR using the primers *Leish150* and *Leish152 5* for the detection of the kDNA of *Leishmania* sp.^6^. PCR of the *gapdh* housekeeping gene was performed using the negative (no-template) control. DNA extraction from tissue and blood samples was performed using the Puregene DNA extraction kit (Qiagen). The presence of *Leishmania* was verified by PCR-based amplification of a fragment of *ITS1* gene (300-350 bp)^7^ ^-^ ^8^, and semi-nested PCR for a fragment of *hsp70* (640 bp) (new primers designed by one of us, PHFS, unpublished). Amplification of the mammalian *cytb* gene^9^ was used as an internal control to confirm the quality of the extracted DNA. Reference strains of *Leishmania* from the Grupo de Estudos em Leishmanioses were used as positive controls for the reactions. Positive samples for *ITS1* and *hsp70* were sequenced on ABI 3730 analyzer (Applied Biosystems), and the sequences were identified by the GenBank BLAST (http://blast.ncbi.nlm.nih.gov/). The *cytb* fragment of *Proechimys* was sequenced to determine the host to species level using the same methods described above.

No dog samples were positive for the presence of anti-*Leishmania* antibodies (anti-rK28 for DPP® and anti-*Leishmania major*-soluble antigen for ELISA) or the presence of parasite DNA, suggesting that these animals do not play an important role in the *Leishmania* cycle inside the WIT.

Of the samples collected from animals hunted by the villagers, 86 samples (tissue and blood) obtained from 63 individuals of 24 different species were analyzed. Two rodent samples, one from an agouti *Dasyprocta* sp. and another from the spiny rat *Proechimys cuvieri* (GenBank accession number MK585528), were positive for *Le. infantum*. The positive sample from *Dasyprocta* sp. was from a hunted animal in the village of Kanikani, in an area of primary forest near the limit of the WIT (*Le. infantum* GenBank accession number MK585526). The sample from *P. cuvieri* was collected in the peridomicile of the village of Boa Vista, but *hps70* sequence could not be deposited in GenBank due to its small size (<200 bp). To the best of our knowledge, this is the first report of natural *Le. infantum* infection in *P. cuvieri.*

*Dasyprocta* spp. are considered important hosts of *Leishmania amazonensis* ^4^ *. Leishmania infantum* has already been detected in the rodent *Dasyprocta azarae* ^4^ *.* Caviomorphs from the genus *Proechimys* have also previously been found to be infected with various *Leishmania* species. These rodents are characterized by their longevity (more than 3 years in captivity) and high abundance in most localities where they are found, within the tropical forests of Central and South America^10^. Various *Proechimys* species have been identified as potential reservoirs of *L. amazonensis* in Brazil and French Guiana, as demonstrated by their frequent skin parasitism, as confirmed by tissue culture^11^ ^-^ ^12^. For example, in French Guiana, this infection was observed in two sympatric species, *P. cuvieri* and *Proechimys guyanensis* ^13^. Other reports of natural infection of these rodents include the following: *Leishmania (Viannia)* spp. in *Proechimys semispinosus* from Colombia^14^ and *Leishmania guyanensis* in *Proechimys* sp*.* from French Guiana^12^and Brazil^15^.

Despite the wide distribution of *Le. infantum* in Brazil, this is the first record of infection with this parasite in wild vertebrate hosts in the State of Amapá. Considering that sampling occurred in 2015, this would be the first detection of the parasite in the State of Amapá. Therefore, a wild sylvatic cycle of *Le. infantum* appears to have existed in the central region of the State of Amapá in 2015, before the first reported domestic canine cases of AVL in the state in 2017. Further research is required to investigate the presence of *Le. infantum* in wild vertebrate hosts and to determine the origin and endemicity of sylvatic transmission cycles, whether they are self-limited acquisitions from domestic reservoirs or stable long-term enzootic reservoirs of parasite transmission.
